# Combined neuromodulatory interventions in acute experimental pain: assessment of melatonin and non-invasive brain stimulation

**DOI:** 10.3389/fnbeh.2015.00077

**Published:** 2015-03-31

**Authors:** Nádia Regina Jardim da Silva, Gabriela Laste, Alícia Deitos, Luciana Cadore Stefani, Gustavo Cambraia-Canto, Iraci L. S. Torres, Andre R. Brunoni, Felipe Fregni, Wolnei Caumo

**Affiliations:** ^1^Post-Graduate Program in Medical Sciences, School of Medicine, Universidade Federal do Rio Grande do Sul (UFRGS)Porto Alegre, Brazil; ^2^Pain and Anesthesia in Surgery Department, School of Medicine, Universidade Federal do Rio Grande do SulPorto Alegre, Brazil; ^3^Pharmacology Department, Instituto de Ciências Básicas da Saúde, Universidade Federal do Rio Grande do SulPorto Alegre, Brazil; ^4^Berenson-Allen Center for Noninvasive Brain Stimulation, Department of Neurology, Beth Israel Deaconess Medical Center, Harvard Medical SchoolBoston, USA; ^5^Service of Interdisciplinary Neuromodulation, Department and Institute of Psychiatry, University of São PauloSão Paulo, Brazil; ^6^Pain and Palliative Care Service at Hospital de Clínicas de Porto Alegre (HCPA), Laboratory of Pain and Neuromodulation at UFRGSPorto Alegre, Brazil

**Keywords:** tDCS, TMS, CPM, pain threshold, melatonin, clinical trial

## Abstract

Transcranial direct current stimulation (tDCS) and melatonin can effectively treat pain. Given their potentially complementary mechanisms of action, their combination could have a synergistic effect. Thus, we tested the hypothesis that compared to the control condition and melatonin alone, tDCS combined with melatonin would have a greater effect on pain modulatory effect, as assessed by quantitative sensory testing (QST) and by the pain level during the Conditioned Pain Modulation (CPM)-task. Furthermore, the combined treatment would have a greater cortical excitability effect as indicated by the transcranial magnetic stimulation (TMS) and on the serum BDNF level. Healthy males (*n* = 20), (aged 18–40 years), in a blinded, placebo-controlled, crossover, clinical trial, were randomized into three groups: sublingual melatonin (0.25 mg/kg) + a-tDCS, melatonin (0.25 mg/kg) + sham-(s)-tDCS, or sublingual placebo+sham-(s)-tDCS. Anodal stimulation (2 mA, 20 min) was applied over the primary motor cortex. There was a significant difference in the heat pain threshold (°C) for melatonin+a-tDCS vs. placebo+s-tDCS (mean difference: 4.86, 95% confidence interval [CI]: 0.9 to 8.63) and melatonin+s-tDCS vs. placebo+s-tDCS (mean: 5.16, 95% CI: 0.84 to 8.36). There was no difference between melatonin+s-tDCS and melatonin+a-tDCS (mean difference: 0.29, 95% CI: −3.72 to 4.23). The mean change from the baseline on amplitude of motor evocate potential (MEP) was significantly higher in the melatonin+a-tDCS (−19.96% ± 5.2) compared with melatonin+s-tDCS group (−1.36% ± 5.35) and with placebo+s-tDCS group (3.61% ± 10.48), respectively (*p* < 0.05 for both comparisons). While melatonin alone or combined with a-tDCS did not significantly affect CPM task result, and serum BDNF level. The melatonin effectively reduced pain; however, its association with a-tDCS did not present an additional modulatory effect on acute induced pain.

**Trial registration**: current controlled trial is registered at clinical trials.gov upon under number: NCT02195271.

## Introduction

Transcranial direct current stimulation (tDCS) is capable of modulating pain systems. Several studies have shown that tDCS applied on the primary motor cortex (M1) and/or the prefrontal cortex (among others) shows clinically significant pain reduction in various chronic pain syndromes, such as fibromyalgia (Fregni et al., 2006; Lefaucheur et al., 2008; Valle et al., 2009; Mendonca et al., 2011; Mylius et al., 2012; O’Connell et al., 2014; Vaseghi et al., 2014), Phantom pain (Bolognini et al., 2013), trigeminal neuralgia (Hagenacker et al., 2014), chronic migraine (Dasilva et al., 2012), low back pain (O’Connell et al., 2013) and Myofascial Pain Syndrome (Sakrajai et al., 2014) tDCS was shown to have a benefit on decreasing pain scores. However, these positive results should not be considered in isolation as some of these studies have methodological shortcomings that may bias these results, leading for instance to false positive findings. The proposed mechanism for tDCS on pain rests on its polarity-dependent shifts of the resting membrane potential and consequent cortical and subcortical modulation (Simis et al., 2014). Hence, one strategy to optimize the analgesic effects of active (a)-tDCS is its combination with pharmacological interventions (Brunoni et al., 2011c).

Which has shown advantages such as the augmentation of its clinical effects, as was observed when combined with sertraline for major depression (Brunoni et al., 2013). In pain, a case report of tDCS combined with D-cycloserine (an *N*-methyl-D-aspartate agonist) suggested its beneficial clinical effects (Antal and Paulus, 2011).

Pre-clinical evidence have demonstrated melatonin effects on inflammatory (Laste et al., 2013) and neuropathic pain (Ambriz-Tututi and Granados-Soto, 2007), and clinical trials in acute (Caumo et al., 2007, 2009) and chronic human pain (Citera et al., 2000; Hussain et al., 2011; Schwertner et al., 2013; Vidor et al., 2014). Melatonin modulates pain systems such as the gamma-aminobutyric acid (GABAergic) and opioidergic systems (Ambriz-Tututi and Granados-Soto, 2007; Zurowski et al., 2012). Its long-term use in endometriosis and fibromyalgia improves pain and decreases the levels of serum brain-derived neurotrophic factor (BDNF; Schwertner et al., 2013; de Zanette et al., 2014). Furthermore, an experimental study showed that melatonin constrained the synaptic plasticity in a concentration-dependent manner (≥1 nM) (Wang et al., 2005), affecting networks that are not directly influenced by tDCS, such as subcortical pain circuits. The concurrent use of tDCS and conditioned pain modulation (CPM), which modulates the descending pain control systems, also show a synergistic effect (Reidler et al., 2012); thus, it is conceivable that such a combination would potentiate melatonin’s effects on pain.

Plasticity in both excitatory and inhibitory circuits in the human motor cortex is regulated by homeostatic metaplasticity (Murakami et al., 2012). Therefore, in this explanatory trial, we tested the hypothesis that compared to the control condition and melatonin alone, a-tDCS combined with melatonin would have a greater effect on pain modulatory effect, as assessed by quantitative sensory testing (QST) and by the pain level during the CPM-task. Furthermore, the combined treatment would have a greater cortical excitability effect as indicated by the transcranial magnetic stimulation (TMS) and on the serum BDNF level.

## Material and Methods

### Study Design, Setting, and Participants

All volunteers provided written informed consent before participating in this study, and the protocol was approved by the Research Ethics Committee at the Hospital de Clínicas de Porto Alegre (Institutional Review Board IRB -13-0155) according to the Declaration of Helsinki. The volunteers were recruited from the general population by advertisement postings in the universities, on the internet, and in public places in the Porto Alegre area. Subjects were considered eligible to participate if they were male, right-handed, and between 19 and 40 years of age, and were screened for eligibility by phone. They answered a structured questionnaire that assessed the following variables: current acute or chronic pain conditions, use of analgesics in the past week, rheumatologic disease, clinically significant or unstable medical or psychiatric disorder, history of alcohol or substance abuse in the past 6 months, neuropsychiatric comorbidity, and use of psychotropic drugs. Subjects responding affirmatively to any of these questions, and those with contra-indications for TMS (Rossi et al., 2009) were excluded. Subjects with Beck Depression Inventory (BDI; Warmenhoven et al., 2012) scores higher than 13 were also excluded (Beck et al., 1996). We include males only to exclude the influence of the cyclical fluctuation of gonadal steroids during the menstrual cycle on pain threshold and in the cortical excitability parameters (Smith et al., 2002; Stefani et al., 2012).

### Sample Size

The number of subjects in each study group was determined according to parameters of a previous study (Stefani et al., 2012). A priori estimate indicated that in a superiority test from a cross-over design, a sample size of 20 subjects divided into three groups with a 2:2:1 ratio, in three sessions, to test for difference between interventions groups on mean of 2.5°C (SD 3°C) for the heat pain threshold (HPT), with a variation coefficient of 0.5, superiority margin 0.22 and to achieves 80% power at a 5% significance. We estimated a sample size for a large effect size using the Analysis of variance. To be an incomplete blocks crossover trial each subject received some interventions but not all subjects received interventions in the third session. This mean that the allocation in a cross-over manner in the first and second sessions was a-tDCS+melatonin (*n* = 8), s-tDCS+melatonin (*n* = 8) and in s-tDCS+placebo (*n* = 4), respectively. In the third session a-tDCS+melatonin (*n* = 4), s-tDCS+melatonin (*n* = 4) and in s-tDCS+placebo (*n* = 2), respectively. The estimative was determined using the Power Analysis and Sample Size Software PASS version 13 (NCSS Statistical Software, Kaysville, Utah).

### Interventions

The intervention involved one dose of sublingual melatonin (Sigma Chemical, Germany; batch-by-batch certificates of analysis for authenticating the purity of each batch provided): 0.25 mg/kg (maximum dose 20 mg), or placebo (Stefani et al., 2013). This solution was combined with 0.5 mL of 10% glucose solution. The placebo was an equivalent volume of 10% glucose solution. The tDCS was introduced 10 min after administer the melatonin to conclude the session (20 min) 30 min after administer the melatonin, since in previous study we demonstrated that at this time we get a serum peak of melatonin when it is used sublingual (Stefani et al., 2013).

tDCS is a therapeutic tool that is relatively inexpensive, non-invasive, painless, safe, its shape can be simulated (sham) and used efficiently for double-blinded studies. In this study, the anode was positioned over the left M1, and the cathode was positioned on the right supraorbital region. The rubber electrodes were inserted into a 35-cm^2^ sponge (moistened with NaCl). The current was 2 mA, and the attachment of electrodes to the scalp was maintained by an elastic band (Vandermeeren et al., 2010). The stimulation time was 20 min, consistent with previous studies (Valle et al., 2009; Knotkova et al., 2013). For the sham conditions, the device was turned off after 1 min of starting the stimulation, which is a reliable blinding method (Brunoni et al., 2011b), capable of mimicking the common adverse effects induced by the real stimulation (Brunoni et al., 2011a). The evaluators and subjects were blinded to the treatment; contact between participants was avoided to enhance study blinding.

### Randomization

**Figure 1:**
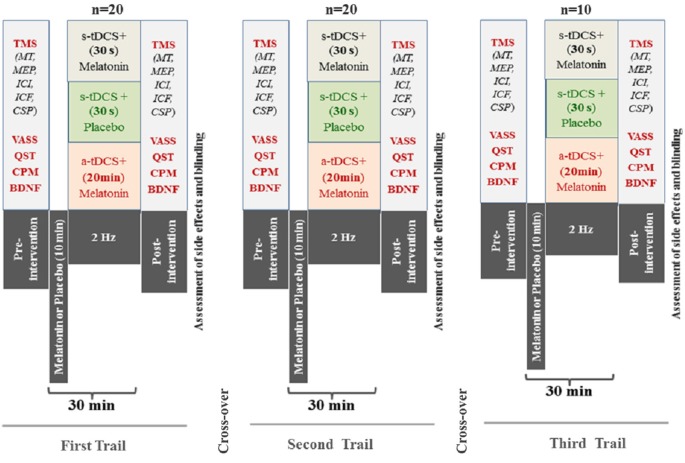
**Experimental desing—Cross-over, assessements and interventions in each one of three sessions**. The period between each session was 1 week. (Motor evoked potential: MEP); Intra-cortical inhibition (ICI) expresses the relationship between the amplitude of wave and motor evoked potentials (relative amplitude, express in %), at inter-stimuli intervals (ISIs) of 2 ms with paired-pulse. The first is a sub-threshold stimulus [80% of the rest motor threshold (rMT)] followed by the second one which is a suprathreshold stimulus (130% rMT). Cortical silent period (CSP) expressed in milliseconds (ms); Motor-evoked potentials (MEP) expressed in mV, evoked by a stimulus of 130% the intensity of the rMT, and should have peak-to-peak MEP amplitude of at least 1 mV. Conditioned Pain Modulation (CPM); Visual Analogue Sleepiness Scale (VASS); Brain Deriavete Neural Factor (BDNF).

The randomization was generated by a computer with a fixed block size of 5. Twenthy subjects were randomly allocated to receive three sequences of treatment (melatonin+active(a)-tDCS, melatonin+sham(s)-tDCS, and s-tDCS+placebo). An allocation of 2:2:1 in favor of the melatonin treatment to maximize allocation to the experimental group and to improve the experimental power. To be an incomplete blocks crossover trial each subject received some interventions but not all subjects received interventions in the third session. This mean that the allocation in a cross-over manner in the first and second sessions was a-tDCS+melatonin (*n* = 8), s-tDCS+melatonin (*n* = 8) and in s-tDCS+placebo (*n* = 4), respectively. In the third session a-tDCS+melatonin (*n* = 4), s-tDCS+melatonin (*n* = 4) and in s-tDCS+placebo (*n* = 2), respectively. The experimental desing and interventions in each session is presented in the Figure 1. Before the recruitment phase, opaque envelopes containing the protocol materials were prepared. Each opaque envelope was sealed and numbered sequentially. The opaque envelopes were opened by the nurse who administered the medications only after gaining subjects’ informed and signed consent.

### Blinding

To control for possible measurement bias participants were instructed to discuss all aspects related to their tDCS treatment only with their treating physician (rather than the research personnel). During the sham stimulation, subjects underwent tDCS experiences that were comparable to the active stimulation. Individuals other than those responsible for administering the interventions were blinded to the allocated interventions. Further, to assess whether blinding was effective, at the end of the experiment we asked participants to guess whether they had received active or sham tDCS and to rate their confidence on the answer on a Likert scale with five categories (no confidence to completely confident). This scale was used to assess the blinding about both interventions (tDCS and sublingual).

### Outcomes

The primary outcome was the HPT as assessed by QST. The secondary outcomes were the excitability of the cortical spinal system indexed by motor-evoked potentials (MEPs), pain reduction on the Numerical Pain Scale (NPS_0–10_) during the CPM task, and other cortical excitability parameters (intra-cortical facilitation (ICF), current silent period (CSP), intra-cortical inhibition (ICI)), and serum BDNF.

The method of limits with a computer Peltier-based device thermode (30 × 30 mm) was used to assess the heat pain threshold (HPT; Schestatsky et al., 2011). The thermode was attached to the skin on the ventral aspect of the mid-forearm, and the temperature was increased at a rate of 1°C/s, from 32°C to a maximum of 52°C, which primarily stimulates C-nociceptive afferents (Backonja et al., 2013). Participants were asked to press a button as soon as the sensation of heat began (heat detection threshold) and as soon as the stimulation became painful (HPT). Three assessments were performed with an inter-stimuli interval of 40 s. Each subject’s HPT was defined as the mean painful temperature of the three assessments. The position of the thermode was slightly altered between trials (although it remained on the left ventral forearm) to avoid either sensitization or response suppression of the cutaneous heat nociceptors. The same equipment was used to determine the maximum tolerated temperature, where volunteers pressed a button to stop the temperature increase. If 52°C was achieved before reporting pain, the device cooled down automatically and the pain threshold was considered unknown.

To test the CPM, we used the term CPM rather than diffuse noxious inhibitory control/DNIC because of the recent recommendations of Yarnitsky et al. (2010), we used the protocol of Tousignant-Laflamme et al. (2008) and the guidelines for the cold-pressor task (CPM-TASK) as an experimental pain stimulus (von Baeyer et al., 2005). The CPM-TASK activates the diffuse noxious inhibitory control-like effect (CPM) because it is a strong nociceptive stimulus that takes place over a lengthy time span (Willer et al., 1989) and is applied over a large body surface area (Marchand and Arsenault, 2002). The CPM-TASK allows us to modify the endogenous pain-modulating system. To quantify the CPM, we evaluated the pain intensity of three tonic heat pain (HPT) test stimuli separated by a CPM-TASK. Although the HPT might lead to habituation and sensitization according to the dual process theory, cold water to zero is a reliable stimulus to induce CPM (Tousignant-Laflamme et al., 2008).

CPM-Task: The cold-pressor task was used as a conditioning stimulus to elicit a strong and prolonged pain sensation to trigger the CPM. The CPM-TASK consisted of immersing the non-dominant hand in cold water (zero to 1°C) for 1 min. During the last 30 s of the cold-water immersion, the HPT procedure was administered over the right forearm (dominant forearm). The temperature was held constant during the experiment for each subject. The HPT that elicited pain ratings of 6/10 on the Numerical Pain Scale [(NPS) 0/10] (HPT_60_) was used for the first HPT before the CPM-TASK (HPT0). After a short break, the HPT0 was applied at the volar region. Following HPT0, the CPM-TASK was used to trigger the CPM. One minute after the CPM-TASK, we applied the second HPT (HPT1). We quantified the amount of the CPM by subtracting the mean pain rating of HPT1 from the first HPT0 before the CPM-TASK (HPT1); negative values indicate inhibitory CPM. This test was applied after measuring the cortical excitability parameters.

Cortical excitability parameters were registered through surface electromyography recordings, which were gathered at the contralateral right first dorsal interosseous muscle using Ag/AgCl electrodes. First, the resting motor threshold (RMT) was determined by obtaining five MEPs with a peak-to-peak amplitude of 50 μV out of 10 consecutive trials using the minimum output of the TMS device. Next, 10 MEPs were recorded with an intensity of 130% of the individual RMT. The CSPs were assessed during muscle activity measured by a dynamometer to be approximately 20% of the maximal force. Accordingly, 10 CSPs were recorded using an intensity of 130% of the RMT. The short-interval ICI (SICI), using an inter-stimulus interval of 2 ms was also assessed. The first conditioning stimulus was set at 80% of the RMT, whereas the second test stimulus was set at 100% of the individual MEP intensity. The ICF was assessed with an inter-stimulus interval of 12 ms. Paired-pulse TMS was conducted in a randomized order for a total of 30 trials (10 for each SICI, ICF, and control stimuli). Off-line analyses included collecting the amplitudes of all MEP, SICI, and ICF values, as well as the duration of the CSPs. The corresponding units for these parameters are mV for MEP, ratio to MEP for SICI and ICF, and ms for CSP (Pascual-Leone et al., 1994).

The serum BDNF concentration was determined using an enzyme-linked immunosorbent assay kit (Chemicon/Millipore, catalog n° CYT306). The serum was frozen at −80°C until the assays were performed.

### Other Instruments and Assessments

Pain catastrophizing thinking was assessed using the validated Brazilian-Pain Catastrophizing Scale (Sehn et al., 2012). Depression symptoms were screened using the BDI (Warmenhoven et al., 2012). Anxiety was measured with the State-Trait Anxiety Inventory (STAI), adapted to Brazilian Portuguese (Kaipper et al., 2010). Demographic data were gathered using a standardized questionnaire. The clinical assessment of sedation was determined by simultaneous recording using a visual analog scale (VAS_0–10_) ranging from zero (sleepiness) to 10 (completely awake). To assess safety, we used the Systematic Assessment for Treatment with tDCS questionnaire based on previously reported adverse events (Brunoni et al., 2011b).

### Statistical Analyses

The differences among the sequence cohort were examined with the analysis of variance (ANOVA) for parametric variables, and categorical outcomes were examined by chi-square or Fisher’s tests.

Continuous data were evaluated for normality using Shapiro-Wilk test. After verifying the corresponding assumptions the results were evaluated using the absolute mean variation for HPT of delta values (post-treatment minus pre-treatment). We analyzed the data using a mixed ANOVA model in which the independent variables were the cohort time of session (time), treatment (placebo+s-tDCS, melatonin+s-tDCS, and melatonin+a-tDCS), the interaction term time vs. the treatment group, and subject identification.

The results were evaluated using the absolute mean variation for MEPs of the percentage of variation [(post-treatment−pre-treatment)/post-treatment] × 100. The HPT was adjusted by the sleepiness score assessed by a VAS_0–10_. All analyses were performed with two-tailed tests at the 5% significance level. All analyses were adjusted for multiple comparisons using Bonferroni test. However due to the excessive number of outcomes, some of our results should be considered exploratory and thus need to be replicated in confirmatory trials. An intention-to-treat analys was planned according to the last observation carried forward through the time points if we had oberved dropouts. The analyses were performed with SPSS version 20.0 (SPSS, Chicago, IL).

## Results

### Subject Characteristics

Twenty healthy subjects were randomized, with the ratio of 2:2:1 to the three interventions, in three sessions to participate in the three sequences of treatment (Figure 1). The demographic and psychological characteristics of the subjects according to the sequence allocation were comparable and are shown in Table 1. All subjects completed the course protocol for which they had been randomized. There was no carry over effect, tested by comparison of pre-treatment assessments (*p* > 0.05). Although participants correctly guessed the intervention used in the transcranial stimulation (tDCS) and sublingual, when the question was about the level of certain of their assigned intervention group, only melatonin was guessed correctly but tDCS not. A maximum of 23% of the subjects in each group correctly guessed the active-tDCS condition; the level of confidence in the intervention was moderate to high in more than 75% of the individuals in all groups, and the percentages of answers between groups, for each item were similar without statistically significant differences (*p* > 0.05, for both measures). Importantly, our results would not change if we exclude subjects with higher incidence of adverse effects.

**Table 1:** **Values are given as the mean (±SD) or as a frequency according to the sequence cohort (*n* = 20)**.

	Sequence trails			
	First (*n* = 20)	Second (*n* = 20)	Third (*n* = 10)	*p*
Age (years)	25.37 (5.39)	25.67 (5.39)	25.60 (5.27)	0.96
Education (years)	16.33 (4.68)	16.56 (4.67)	16.56 (4.65)	0.85
Smoking (yes/no)	1 (yes: 5.26%)	1 (yes: 5.55%)	1 (yes: 5.26%)	0.52
Social alcohol consumption – not more than once a week (yes/no)	8 (yes: 42.05%)	8 (yes: 44.44%)	4 (yes: 43.44%)	0.54
Body mass index	25.38 (3.89)	25.78 (3.24)	25.48 (3.22)	0.33
State-anxiety	19.84 (4.02)	19.89 (3.55)	19.87 (3.87)	0.57
Trait-anxiety	15.92 (3.46)	16.17 (3.91)	15.98 (3.22)	0.94
Depressive symptoms on the Beck Inventory	3.79 (3.38)	2.94 (3.10)	3.02 (3.45)	0.73
Pain Catastrophizing Scale–total score	5.84 (6.82)	7.67 (9.15)	6.44 (7.78)	0.35
Brain-derived neurotrophic factor (ng/ml) before intervention
Placebo+s-tDCS	48.78 (15.04)	46.38 (7.89)	47.58 (10.78)	0.52
Melatonin+s-tDCS	50.69 (16.02)	43.9 (11.95)	46.87 (13.04)	0.89
Melatonin+a-tDCS	50.0 (15.02)	45.21 (11.71)	46.34 (13.04)	0.78
Psychophysical pain testing
Heat pain threshold (HPT) (°C)	42.98 (4.07)	42.7 (3.93)	43.0 (2.77)	0.98
Heat pain threshold 60% (HPT60) (°C)	44.48 (3.19)	45.02 (3.20)	44.75 (1.98)	0.51
Maximal tolerated heat (°C)	44.38 (3.26)	44.97 (3.24)	44.75 (1.89)	0.82

*SD = standard deviation, tDCS = transcranial direct current stimulation*.

The incidence of reported side effects presented a similar distribution between groups. Headache, neck and scalp pain, skin redness, mood changes, and difficulties in concentration were reported by <15% of subjects. Burning and itching were reported by more than 25% of the subjects. Tingling was the most common side effect reported, with an incidence higher than 30%. The scores on the VAS_0–10_ (higher score less sleepiness) showed that placebo+s-tDCS groups 9.62 ± 0.52 induced lower sleepiness than the active arms (melatonin+a-tDCS 5.62 ± 1.31 and melatonin+s-tDCS 5.93 ± 1.43; *p* < 0.01 for each comparison vs. placebo+s-tDCS), although there was no difference between the two active tDCS groups (*p* = 0.9). The VAS_0–10_ scores for sleepiness vs. group comparison did not demonstrate a significant interaction (*F*_(2,46)_ = 0.18; *p* = 0.84). Additionally, there was no statistically significant effect of sleepiness score on HPT (*β* = 0.31, *t* = 1.16; 95% confidence interval [CI]: −0.23 to 0.85; *p* = 0.25).

### Treatment Effects on the HPT (Primary Outcome) and on the Descending Modulatory System (Secondary Outcome)

**Figure 2:**
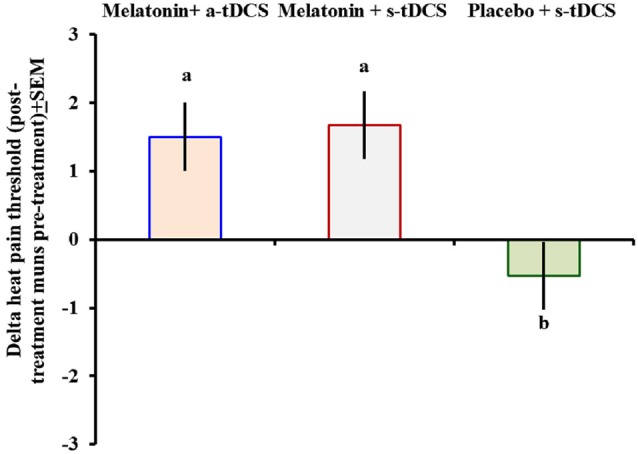
**Heat pain threshold scores before and after intervention presented as delta values (post- minus pre-treatment)**. Error bars indicate the standard error of the mean. The letter *b* indicates a significant difference between the melatonin+a-tDCS group and the melatonin+s-tDCS and placebo+s-tDCS groups (*p* < 0.02). All comparisons were performed using a mixed analysis of variance model, followed by the Bonferroni correction for multiple *post hoc* comparisons. tDCS = transcranial direct current stimulation.

In the incomplete factorial analysis, there were two factors: (a-tDCS and s-tDCS) and melatonin (real or placebo). The analysis showed no significant interaction between tDCS and melatonin on HPT (*F*_(2,46)_ = 0.3; *p* = 0.95), but a significant main effect for treatment was observed (*F* = _(2,46)_ = 3.94; *p* = 0.02); (Table 2). The differences mean in the HPT tests are presented in (Figure 2).

**Table 2:** **The mean delta score (SD) (post-treatment values minus pre-treatment values) of the heat pain thresholds and motor-evoked potentials (*n* = 20)**.

	Pre-intervention	Post-intervention	Mean difference (post-intervention–pre-intervention, 95% CI)	% (SD)*
Heat Pain Threshold (°C) (*primary*)
Placebo+s-tDCS	43.0 (2.77)	42.46 (2.97)	−0.53 (−1.83 to 0.77)	-1.21 (3.52)*^a^*
Melatonin+s-tDCS	42.67 (3.39)	44.34 (3.94)	1.67 (0.7 to 2.64)	3.94 (4.81)*^b^*
Melatonin+a-tDCS	43.0 (3.90)	43.84 (4.28)	1.50 (0.57 to 2.45)	3.65 (5.54)*^b^*
*p* value^¥^	0.02
Motor-evoked potential (mV) (*secondary*)
Placebo+s-tDCS	1.39 (0.19)	1.45 (0.22)	0.06 (−0.36 to 0.22)	4.31 (10.56)*^a^*
Melatonin+s-tDCS	1.76 (0.28)	1.73 (0.43)	−0.03 (−0.16 to 0.02)	-1.36 (5.35)*^a^*
Melatonin+a-tDCS	1.84 (0.4)	1.17 (0.5)	−0.37 (−0.22 to −0.39)	-19.96 (5.2)*^b^*
*p* value^¥^	0.003

*SD = standard deviation, CI = confidence interval, tDCS = transcranial direct current stimulation*.

*Percentage represents the percent change, calculated as [(post-intervention–pre-intervention)/post-intervention] × 100.

*^¥^*p* value represents the results from the mixed-model analysis of variance × group interaction (for the main analysis) and for the factorial analysis*.

*Different superscripts (a and b) indicate significant differences among intervenios groups according to the Bonferroni test*.

The function of the descending modulatory system was assessed using the CPM task. Although all the interventions improved the pain reduction during the CPM task, there were no differences in their effectiveness between them (*p* > 0.05). The reduction in pain scores on the NPS0-10 during the CPM task was 48.41% (HPT0 = 5.04 ± 1.06; HPT1 = 2.60 ± 1.27) in the melatonin+active-tDCS group, 37.88% (HPT0 = 4.25 ± 1.37; HPT1 = 2.64 ± 1.55) in the melatonin+sham-tDCS group, and 33.74% (HPT0 = 4.83 ± 1.06; HPT1 = 3.2 ± 1.42) in the placebo+sham-tDCS group. These results reveal that the interventions did not change the descending modulatory system as assessed by the CPM task.

### Effect on the Neurophysiological Outcomes (Secondary), as Indicated by the TMS Cortical Excitability Parameters: MEPs, ICI, ICF, CSP, and BDNF

**Figure 3:**
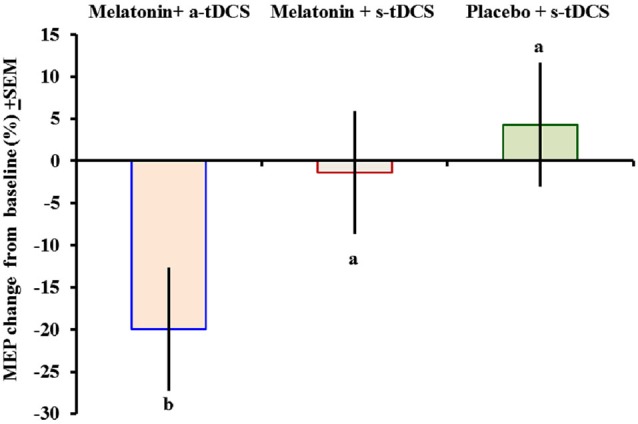
**Motor-evoked potential (MEP) changes from baseline presented as percentages (post intervention minus pre-intervention)**. A letter *b* indicates a significant difference between the melatonin+a-tDCS group and the melatonin+s-tDCS and placebo+s-tDCS groups (*p* < 0.05). All comparisons were performed using a mixed analysis of variance model, followed by the Bonferroni correction for multiple *post hoc* comparisons. tDCS = transcranial direct current stimulation.

Similar analyses showed significant main effects of the intervention group for MEPs (*F*_(2,46)_ = 11.55; *p* = 0.03). There was significant difference in MEP amplitude between the treatment group melatonin+a-tDCS and the melatonin+s-tDCS group (−19.96% ± 5.2 vs. −1.36% ± 5.35; mean difference: −18.60%, 95% CI: −42.44 to −7.12; *p* = 0.03) and melatonin+a-tDCS and the placebo+s-tDCS group (−19.96% ± 5.2 vs. 3.61% ± 10.48; mean difference: −23.57%, 95% CI: −39.68 to −1.2; *p* = 0.01). However, there was no significant difference in MEP amplitude between the melatonin+s-tDCS and the placebo+s-tDCS group (−1.36% ± 5.35 vs. 4.31% ± 10.56; mean difference: −5.67%, 95% CI: −39.68 to −1.2; *p* = 0.48). The differences between the groups in the percentage of variation before and after treatment are shown in Figure 3.

The MEP differences (mean ± SD) before and after treatment, irrespective of the session sequence, are presented in Table 2. Melatonin alone did not result in any significant MEP changes.

The effects of the interventions on the secondary outcomes related to cortical excitability are presented in Table 3. The interventions did not induce significant changes on the other cortical excitability parameters (ICF, ICI, and CSP). No significant difference between the treatment groups was observed for the serum BDNF levels at baseline, which had great variability (Table 1). From the baseline level, the serum BDNF level demonstrated a mean decrease of 10.96% in the placebo+s-tDCS group, whereas the melatonin+s-tDCS and melatonin+a-tDCS groups presented mean reductions of 12.79% and 6.09%, respectively (Table 3).

**Table 3:** **Outcomes related to cortical excitability and serum BDNF (20 subjects) and the number of assessment**.

Intervention	Mean (SD)	*p*	SDM
**Dependent variable: resting motor-threshold**
Placebo+sham-tDCS (*n* = 10)	41.10 (5.70) vs. 40.07 (5.83)*^a^*	0.69	0.18
Melatonin+sham-tDCS (*n* = 20)	41.56 (6.54) vs. 42.56 (5.65) *^a^*		0.15
Melatonin+active-tDCS (*n* = 20)	39.11 (6.72) vs. 40.05 (6.20) *^a^*		0.13
**Dependent variable: cortical silent period**
Placebo+sham-tDCS (*n* = 10)	64.16 (9.8) vs. 63.95 (10.76) *^a^*	0.93	−0.02
Melatonin+sham-tDCS (*n* = 20)	64.51 (20.13) vs. 65.65 (20) *^a^*		−0.06
Melatonin+active-tDCS (*n* = 20)	62.08 (21.25) vs. 63.02 (23.38) *^a^*		−0.05
**Dependent variable: intra-cortical facilitation**
Placebo+sham-tDCS (*n* = 10)	1.07 (0.20) vs. 0.99 (0.20) *^a^*	0.72	0.4
Melatonin+sham-tDCS (*n* = 20)	1.03 (0.17) vs. 1.07 (0.20) *^a^*		−0.24
Melatonin+active-tDCS (*n* = 20)	1.08 (0.33) vs. 1.05 (0.24) *^a^*		0.09
**Dependent variable: intra-cortical inhibition**
Placebo+sham-tDCS (*n* = 10)	0.22 (0.19) vs. 0.21 (0.09) *^a^*	0.32	0.05
Melatonin+sham-tDCS (*n* = 20)	0.26 (0.15) vs. 0.29 (0.12) *^a^*		−0.2
Melatonin+active-tDCS (*n* = 20)	0.23 (0.10) vs. 0.25 (0.11) *^a^*		−0.2
**Dependent variable: serum BDNF ng/ml**
Placebo+sham-tDCS (*n* = 10)	46.76(13.02) vs. 44.25 (12.66) *^a^*	0.48	0.19
Melatonin+sham-tDCS (*n* = 20)	49.19 (14.42) vs. 45.22(11.18) *^a^*		0.27
Melatonin+active-tDCS (*n* = 20)	48.82 (14.08) vs. 46.19 (12.53) *^a^*		0.18

*Mean (SD) pre vs. post-intervention*.

*SD = standard deviation, tDCS = transcranial direct current stimulation*.

*Standardized mean difference (SMD) [(pre minus post)/baseline standard deviation]. The size effect was interpreted as follows: small, 0.20; moderate, 0.50–0.60 and large, 0.80*.

*All comparisons between the melatonin+tDCS, melatonin+sham-tDCS, and placebo+sham-tDCS groups were performed using a mixed analysis of variance model (p > 0.05 for all comparisons). Different superscripts a indicate absence of significant differences among treatment groups according to the Bonferroni test*.

## Discussion

The main findings of this study confirm that melatonin significantly affects the pain pathways, which are not changed by the concurrent tDCS stimulation. Furthermore, this effect does not seem to be associated with changes in cortical excitability. This finding contrasts to our initial hypothesis that melatonin combined with tDCS would improve pain control, considering that treatment with tDCS (Valle et al., 2009; Mendonca et al., 2011) or melatonin alone demonstrated an effect on pain in pre-clinical (Laste et al., 2012a,b), experimental (Stefani et al., 2013), and clinical studies (Caumo et al., 2002, 2007, 2009; Schwertner et al., 2013; Vidor et al., 2013, 2014) One possible explanation for this result is that melatonin induced maximum homeostatic control to modulate the painful stimuli via neurobiological systems that are common targets for both interventions (i.e., melatonin induced a ceiling effect on pain). This hypothesis is biologically plausible and is supported by pre-clinical evidence indicating that the GABAergic (Wilhelmsen et al., 2011), opioid, and glutamatergic systems (Mantovani et al., 2006) act as targets for both melatonin and tDCS.

Another explanation for the lack of an effect when tDCS was combined with melatonin may be that melatonin blocked the effects of tDCS. It has been shown that pharmacological agents (Liebetanz et al., 2002) such as benzodiazepines, are capable of partially blocking the clinical effects of tDCS (Brunoni et al., 2013). Thus, the increased excitability of GABA-A and GABA-B circuits in M1 might increase the inhibitory tone, which is responsible for the general occlusion of the subsequent induction of long-term potentiation- and long-term depression-like plasticity (Castro-Alamancos and Borrell, 1995; Castro-Alamancos et al., 1995; Hess et al., 1996). Therefore, it is plausible that the failure of additive effect of a-tDCS+melatonin is explained by a similar response, because of melatonin action on GABA-A receptor (Coloma and Niles, 1988; Niles and Peace, 1990). It is also possible that the lack of interaction effect is a result of metaplasticity, i.e., when two plasticity protocols are used together, the effect of the first one modulates that of the second (Murakami et al., 2012). Other mechanism to explain this finding is the depotentiation, which refers to two protocols that when used alone do not induce changes in the excitability, but when used together cancel out the effect of a preceding potentiation protocol to achieve homeostasis (Froc et al., 2000; Yashiro and Philpot, 2008; Müller-Dahlhaus and Ziemann, 2014). Accordingly, the tonic depression of the nociceptive threshold may result from the activation of pro-nociceptive areas of the brain or from inhibition of the endogenous pain inhibitory system (Burkey et al., 1999).

Other explanations for these results, that challenge our hypothesis, are evidences of previous clinical studies, which demonstrated that the behavioral data did not indicate a pain-reducing effect of anodal stimulation (Grundmann et al., 2011; Jürgens et al., 2012; Luedtke et al., 2012). Interestingly, previous studies on experimental pain using the same stimulation paradigm also showed inconclusive effects of tDCS on psychophysical variables (Grundmann et al., 2011; Jürgens et al., 2012; Luedtke et al., 2012). These inconclusive effect of change on cortical nociceptive processing, as a response to heat pain was also reported in other recent study, which did not found neither cathodal nor anodal tDCS effect over the left M1 (1 mA, 15 min) (Ihle et al., 2014).

The effect of a-tDCS on neurophysiological outcomes (such as evoked potentials) demonstrated in the present study, were also reproduced in the majority of trials after tDCS (Matsunaga et al., 2004; Csifcsak et al., 2009; Luedtke et al., 2012). Perhaps the psychophysical variables depend on a range of different pathways because evaluation of pain is a more complex process than mere somatosensory processing in evoked potentials. Higher stimulation intensities, longer stimulation duration, or repeated stimulation sessions may be required to produce a statistically significant experimental pain reduction that matches the effect observed in chronic clinical pain studies. Although M1 excitability is a reliable marker for indexing the effects of interventions on pain (Volz et al., 2013; Dall’Agnol et al., 2014; Vidor et al., 2014), this marker seems to be more specific for chronic pain than for acute experimental pain. It also suggests that melatonin modulation on pain does not involve a direct effect on M1, while tDCS does (Reidler et al., 2012; Knotkova et al., 2013).

In addition, we have shown that melatonin’s effect on pain is not mediated by descending pain control systems. In fact, in this study, using the CPM task, the pain score on the NPS_0–10_ was reduced by more than 30% in all of the treatment groups including the control group. This is consistent with previous studies demonstrating an approximated CPM effect of 29% (Pud et al., 2005, 2009; Niesters et al., 2013). The conditioning stimulus used in this study (hand placed in water at 0–1°C for at least 30 s) was a strong, painful stimulus that depresses the nociceptive messages elicited from remote localized body areas. Here, ceiling effects were also possible, i.e., the CPM responses were at their maximum effect given the intensity of the conditioning stimulus used. However, other studies have shown that it is possible to modulate CPM using melatonin, if used in the long-term (de Zanette et al., 2014), or with tDCS alone (Reidler et al., 2012). These results, namely the lack of melatonin-induced M1 modulation and descending inhibitory pain system involvement, support to some extent the notion that acute melatonin after-effects may have limited impact on cortical and spinal systems, thus suggesting that melatonin may modulate subcortical centers. However, this hypothesis needs to be confirmed in further trials with other neurophysiological techniques or functional imaging techniques, such as quantitative electroencephalography, near-infrared spectroscopy (NIRS) or functional magnetic resonance imaging (fMRI).

The a-tDCS effect on the corticospinal system is related to increased MEP amplitude (Table 2), an effect that is consistent with previous studies that the anodal tDCS over the M1 induced an enhancement of the corticospinal excitability (Pellicciari et al., 2013). The tDCS effect on the cortico-subcortical networks is also supported by recent evidence of a functional coupling increase on the thalamo-cortical circuits following anodal stimulation over the motor cortex (Polanía et al., 2012). We speculate that the not site-limited cortical excitability increase could be determined by a decrease of the contralateral hemisphere inhibition, mediated, at least partially, by the anodal tDCS-induced reduction of GABA concentration (Stagg et al., 2009). Also, the tDCS might induce an increased cortical evoked response with a probable concurrent involvement of the N-Methyl-D-aspartate (NMDA) receptors (Islam et al., 1995; Nitsche et al., 2003). Thus, these findings suggest that the modulatory effects produced by a-tDCS were not limited to the targeted cortical area but also occur at distant interconnected sites including spinal tract. Given the results, it is likely that the tDCS does not have a direct excitatory or inhibitory effect but mostly a modulation role, presumably expressed as to changes in the excitability of cortical circuits.

The present findings showed that the use of melatonin alone or with tDCS did not induce changes in the serum BDNF levels. Although it is widely distributed in the CNS, the BDNF has been used as a possible neuroplasticity marker that is modulated by rTMS (Dall’Agnol et al., 2014), tDCS (Brunoni et al., 2014), or melatonin treatment (Schwertner et al., 2013; de Zanette et al., 2014), particularly when assessed in the long-term treatment of chronic pain. However, in this study, serum BDNF was measured in healthy subjects and only a short time after one intervention session. Another possible explanation of the lack of changes in serum BDNF in the present study is that the intervention effect was not sufficient to induce a level of neuroplasticity detectable on serum BDNF. These hypotheses are plausible considering that BDNF is produced in the CNS and transported through the blood-brain barrier via saturable systems (Poduslo and Curran, 1996; Pan et al., 1998; Asmundson et al., 1999). Although the study demonstrated that the CNS contributes to 70−80% of the circulating BDNF (Rasmussen et al., 2009), this measure may underestimate its real level in the CNS, since it was demonstrated that in healthy subjects it can be 14-fold the BDNF level in the plasma (Yoshimura et al., 2014). These findings are important for understanding the physiological mechanisms and the pharmacological and non-pharmacological substrates of the combined effect of melatonin and active-tDCS on pain. However, for a better comprehension this effect further studies are needed in patients with chronic pain, which show amplified sensory pain.

It is important to emphasize that we chose a cross-over design as to have a single-subject design, in which the subjects serve as their own control. This design is sensitive to individual organism’s differences allowing better assessment of causal relationship between the independent and dependent variables (Xeniditis et al., 2001; Dallery et al., 2013). Whereas it reduces the between subjects comparison, it is an ideal strategy to validate results because subjects have significant variability when assessing outcomes related to behavior and physiological parameters. In addition this design also helps with controlling between-subject differences in the effects of stimulation as recent evidence based computational models suggest that inter individual differences in head anatomy may affect the distribution of the electric field in the brain and that a uniform dose of stimulation for all patients may not be the most efficient procedure (Datta et al., 2012). Given the costs associated with individual modeling required to customize the stimulation on an individual basis, our design controls for this issue as we compare the same subjects before and after each intervention (Datta et al., 2012).

Several issues concerning the design of our study must be address:*** First***, the absence of a group of placebo plus a-tDCS is a limitation of our study. However, previous studies showed the effectiveness of a-tDCS in increasing on sensory and pain thresholds in healthy individuals and pain levels in patients with chronic pain (Vaseghi et al., 2014). Other possibility to explain this finding is that s-tDCS potentiates the mechanisms involved in placebo analgesia as suggested by a recent study (Dossantos et al., 2014).*** Second***, even though the tDCS is an efficient technical solutions to conduct blinded studies of both the patients and experimenters (Gandiga et al., 2006) the efficacy of patient blinding has been questioned especially be present at stimulation intensities of 2 mA compared with lower intensities (O’Connell et al., 2012). However, it is improbable that the unblinding change the directions of our conclusions, because the findings did not change when analyzing only subjects that did not guess the allocation group. Accordingly, as neurophysiological studies have shown a stimulation shorter than 3 min induce no significant after–effects (Nitsche and Paulus, 2000). ***Third***, we included only males subjects, because an enhanced pain response in females has been attributed to physiological and psychological variables, including mechanisms of endogenous inhibition, the capability to endure pain, genetic factors, pain expectation and personality traits (Keefe et al., 2000; Wiesenfeld-Hallin, 2005). In this context, the gender may be an important confounding factor because female are more prone to activation upon negative emotional responses (i.e., stress, fear, and anxiety) and higher trait-anxiety is associated with an imbalance between excitatory and inhibitory descending systems of the corticospinal tract (Vidor et al., 2014). Another factor to consider is the hormonal variation throughout the menstrual cycle. Finally, even after the adjustment for multiple comparisons the effect of melatonin was significantly on heat pain threshold and the a-tDCS on MEP (see Table 2). We agree that our study, as the majority of similar studies has also an exploratory nature and thus it is possible that our study has increased type I and type II error. Hence the results of secondary outcomes should be interpreted as explanatory.

The melatonin effectively reduced pain; however, its association with a-tDCS did not present an additional modulatory effect on acute induced pain. Melatonin effects on induced acute pain did not seem to be mediated by cortical or brainstem modulation given the lack of results from the cortical excitability and descending pain control systems. Although the a-tDCS changed the cortical-spinal excitability assessed by MEP this effect not changed the CPM. In fact, these findings might have physiological implications to support an understanding of the maximum homeostatic physiological control when are used combined interventions which have common targets to modulate the painful stimuli.

## Author Contribution

AB participated in the sequence alignment and drafted the manuscript. MT participated in the sequence alignment. ES participated in the design of the study and performed the statistical analysis. FG conceived the study, participated in its design and coordination and helped drafting the manuscript. Nadia Regina Jardim da Silva: nadia.jardimsilva@gmail.com; (UFRGS); FG Gabriela Laste: gabrielalaste@gmail.com; (UFRGS); MT Alícia Deitos: aliciadeitos@gmail.com; (UFRGS); MT Gustavo Cambraia-Canto: guscanto91@gmail.com; (UFRGS); MT Luciana Stefani: lustefani@terra.com.br; (UFRGS); ES Iraci Torres: iracitorres@gmail.com; (UFRGS); AB Andre R Brunoni: brunoni@usp.br; (São Paulo); AB Felipe Fregni: Fregni.Felipe@mgh.harvard.edu; (Harvard Medical School); ES Wolnei Caumo: caumo@cpovo.net. (UFRGS). Responsible for maintaining the study records; FG.

## Conflict of Interest Statement

The authors declare that the research was conducted in the absence of any commercial or financial relationships that could be construed as a potential conflict of interest.
